# Bridging the gap: how to adopt opportunistic plant observations for phenology monitoring

**DOI:** 10.3389/fpls.2023.1150956

**Published:** 2023-10-04

**Authors:** Negin Katal, Michael Rzanny, Patrick Mäder, Christine Römermann, Hans Christian Wittich, David Boho, Talie Musavi, Jana Wäldchen

**Affiliations:** ^1^ Biogeochemical Integration, Max Planck Institute for Biogeochemistry, Jena, Germany; ^2^ Data Intensive Systems and Visualisation, Technische Universitat Ilmenau, Ilmenau, Germany; ^3^ Faculty of Biological Sciences, Friedrich Schiller University, Jena, Germany; ^4^ German Centre for Integrative Biodiversity Research (iDiv), Halle-Jena-Leipzig, Germany

**Keywords:** citizen science, phenology, phenology monitoring, plant identification app, opportunistic plant observation, German Meteorological Service, DWD

## Abstract

Plant phenology plays a vital role in assessing climate change. To monitor this, individual plants are traditionally visited and observed by trained volunteers organized in national or international networks - in Germany, for example, by the German Weather Service, DWD. However, their number of observers is continuously decreasing. In this study, we explore the feasibility of using opportunistically captured plant observations, collected via the plant identification app Flora Incognita to determine the onset of flowering and, based on that, create interpolation maps comparable to those of the DWD. Therefore, the opportunistic observations of 17 species collected in 2020 and 2021 were assigned to “Flora Incognita stations” based on location and altitude in order to mimic the network of stations forming the data basis for the interpolation conducted by the DWD. From the distribution of observations, the percentile representing onset of flowering date was calculated using a parametric bootstrapping approach and then interpolated following the same process as applied by the DWD. Our results show that for frequently observed, herbaceous and conspicuous species, the patterns of onset of flowering were similar and comparable between both data sources. We argue that a prominent flowering stage is crucial for accurately determining the onset of flowering from opportunistic plant observations, and we discuss additional factors, such as species distribution, location bias and societal events contributing to the differences among species and phenology data. In conclusion, our study demonstrates that the phenological monitoring of certain species can benefit from incorporating opportunistic plant observations. Furthermore, we highlight the potential to expand the taxonomic range of monitored species for phenological stage assessment through opportunistic plant observation data.

## 1 Introduction

In recent years, phenology, the study of recurrent events within a plant’s life cycle, has received increasing public and scientific attention: Growing scientific evidence implies that the timings of certain phenological events, e.g., bud break, flowering, fruiting or leaf senescence, are significantly affected by climate change (Piao et al., 2019; Inouye, 2022). The resulting phenological shifts impact the structure and functioning of ecosystems, ranging from affecting species dispersal and disrupting species interactions to altering the carbon cycle (Cleland et al., 2007; Piao et al., 2019). Therefore, continuous monitoring, assessment and modeling of phenological dynamics is critical to understand how plants respond to a changing world and how this affects processes and functions within ecosystems.

Many methods are available to record plant phenology at different temporal and spatial scales (Katal et al., 2022). At the broadest scale, spectral vegetation indices derived by remote sensing and representing the seasonal dynamics of local vegetation are used to inform global models of landscape-scale dynamics (Cleland et al., 2007; White et al., 2009; Richardson et al., 2013). Additionally, PhenoCams are used to collect ground-based phenological data at a network of sites across the world (Brown et al., 2016; Richardson, 2019). Over time, these networks provide a continuous record of plant greening and senescence. Besides these broad-scale phenological patterns, co-occurring species typically show species-specific responses to changes in climate (Bucher et al., 2018; König et al., 2018), that might be only weakly correlated with remotely sensed data on larger spatio-temporal scales (Badeck et al., 2004; Montgomery et al., 2020).

To study and systematically collect detailed information about phenological events at the level of individual species, phenological networks such as the European Phenology Network (EPN) (van Vliet et al., 2003), the International Phenological Garden network (IPG) (Renner and Chmielewski, 2022), the USA National Phenology Network (USA-NPN) (National Phenology Network, 2022) or the PhenObs network (Nordt et al., 2021), have been established. Usually, the phenophases recorded by them are leaf-out, bud break, initial growth for annual plants, expansion of leaves, first flowering day, appearance of fruits, senescence, and leaf abscission (Koch et al., 2007; Morisette et al., 2009; Denny et al., 2014; Berra and Gaulton, 2021; Nordt et al., 2021).

To capture phenological changes at local scales across larger areas, national authorities often establish and maintain networks dedicated to this purpose (Beaubien and Hamann, 2011; Taylor et al., 2019). For example in Germany, the German Meteorological Service (Deutscher Wetterdienst, DWD) curates and manages a national phenological network of citizen scientists. As a result, a growing long-term dataset based on the observations of thousands of trained, voluntary observers is being collected, covering phenological observations dating back to 1951 (Kaspar et al., 2014). Each observer follows a well-defined protocol ensuring consistency, and contributes spatially highly resolved ground-based data. This detailed coverage of multiple phenological stages of different plant species throughout the growing season can be used to inform or calibrate global vegetation and climate models (Badeck et al., 2004). However, the decline in numbers of volunteer observers within these networks poses a significant threat to the accuracy of data interpolation. According to Yuan et al. (2021), the number of volunteer observers in Germany has decreased from over 2,000 in the early 1980s to less than 1,000 in the early 2020s. Similar trends of decreasing observer numbers have been observed in other networks, including the Austrian phenology network (Yuan et al., 2021). To facilitate annual observations, most species observed by phenology networks are woody or represent herbaceous or grass species which are relevant to agriculture. Only a small subset of observed species represents herbaceous wildflowers.

As with fewer numbers of volunteer phenology observers the collection of systematic phenological records declines, there is a noticeable increase in a new source of data, consisting of opportunistic and unsystematic plant observations, originating from AI-based plant identification applications. With the advent of deep learning methods (Goodfellow et al., 2016), AI-based species identification is reaching accuracy levels comparable to human experts (Wäldchen and Mäder, 2018; Jones, 2020; Villon et al., 2020; Pärtel et al., 2021). With these technologies, anyone without prior knowledge of plant species can easily and accurately identify common vascular plants in the field. The shared ancillary information on time and location turns these mobile observations into an invaluable resource for various monitoring tasks (Bonnet et al., 2020; Mahecha et al., 2021). Despite the popularity of these apps, little is known about their potential to capture phenological information (Puchałka et al., 2022). Initial studies show that plants are mostly observed at specific phenological stages, such as flowering or fruit ripeness (Mäder et al., 2021). This offers a wide range of possibilities for plant phenology monitoring and studying large-scale phenological processes. In this study, for the first time, we show how opportunistic plant observations can be used as input data for phenology interpolation models. We use the observations of 17 species recorded with Flora Incognita; an AI-based plant identification app developed to automatically identify vascular plant species (Mäder et al., 2021). Since the actual observation counts reach their highest numbers in Germany, we chose the interpolation model for climatological and phenological maps currently employed by the German Meteorological Service (Müller-Westermeier (1995); W. Janssen (pers. comm.)) for comparison. Furthermore, we explore if species with distinct phenological patterns also share common biological traits, since users of identification apps might document their plant findings based on different characteristics than the trained DWD observers.

We ask: (1) Can phenological phases of plant species, such as onset of flowering be derived from unsystematic and opportunistic plant observations? (2) How can unsystematic observations be processed in order to use them as input for interpolation models in the same way as targeted and systematic observations collected by trained phenology observers? (3) How do spatially interpolated maps differ between opportunistic and targeted observations, and do plant characteristics have an impact on the interpolation validity?

## 2 Materials and methods

### 2.1 Data

#### 2.1.1 Phenological observations from German Meteorological Service

The German Meteorological Service (DWD) is managing a network of registered and trained voluntary observers who observe individual plants of a specific set of species throughout the year, according to a strict protocol (Zimmermann and Polte-Rudolf, 2013; Kaspar et al., 2014). Each observer is assigned a unique ID that is referred to as “station”, covering a specific observation point. Depending on the species, the number of stations is variable, resulting in a heterogeneous spatial resolution of the data. During vegetation season, the observers are asked to visit their observed individuals at least twice a week, noting the day when certain phenological stages have arrived. The final report lists are handed back to the DWD by the end of the year and are made freely available by the organization (https://opendata.dwd.de/climate/environment/CDC/grids/germany/annual/phenology). We refer to this kind of data as “targeted” as they are collected with the purpose of monitoring phenology following a defined standard protocol. For this study, we considered the DWD reports of 2020 and 2021 of all species for which an interpolated dataset was available that reported data on the onset of flowering, resulting in 17 species available for comparison, including 8 trees, 3 shrubs, and 6 herbaceous species (**Table 1**).

**Table 1:** Observation and station count for FI (Flora Incognita) and DWD (German Meteorological Service) data for 17 species and two years (2020 and 2021).

Species			Stations				Special traits			
	FI records		FI		DWD		Growth forms*	Cultivation*	Flowering season*	Conspicuosmess
	2020	2021	2020	2021	2020	2021				
*Alnus glutinosa*	11,741	10,746	486	482	764	840	tree	wild	pre spring	no
*Artemisia vulgaris agg.*	22,707	22,187	513	539	695	699	herb	wild	mid-summer	no
*Betula pendula*	5,835	3,890	231	173	901	849	tree	wild	spring	no
*Brassica napus*	7,400	1,353	280	286	599	590	herb	cultivated	spring	yes
*Calluna vulgaris*	4,806	5,120	103	122	419	406	shrub	wild	early autumn	yes
*Corylus avellana*	20,345	17,694	644	703	955	962	shrub	wild	pre spring	no
*Fraxinus excelsior*	14,483	12,193	565	542	753	733	tree	wild	spring	no
*Galanthus nivalis*	1,242	4,000	–	226	967	999	herb	wild	pre spring	yes
*Malus sylvestris agg.*	21,752	18,518	631	593	843	857	tree	cultivated	spring	yes
*Prunus avium*	15,965	13,395	90	83	134	130	tree	cultivated	spring	yes
*Robinia pseudoacacia*	14,276	14,276	549	555	723	726	tree	wild	start summer	yes
*Salix caprea*	6,842	7,749	294	398	895	913	tree	wild	pre spring	yes
*Sambucus nigra*	26,743	23,339	743	713	962	964	shrub	wild	early summer	yes
*Secale cereale*	4,063	4,106	157	159	344	346	herb	cultivated	early summer	no
*Taraxacum officinale*	25,878	25,592	707	740	981	988	herb	wild	spring	yes
*Tilia platyphyllos*	7,450	5,539	435	355	849	844	tree	wild	summer	no
*Tussilago farfara*	7,945	13,391	219	453	787	781	herb	wild	pre spring	yes

The first column shows the overall number of observations collected via the FI app in each year. The second column shows the resulting number of stations for both sources of data (see the methods section for a detailed explanation of the stations). The last column shows the selected traits for each species. *Traits compiled from the BiolFlor database (Kühn et al., 2004). Conspicuousness was assessed by the authors.

#### 2.1.2 Flora Incognita records

Flora Incognita (FI) is a freely available mobile application allowing automated, image-based identification of wild flowering plants (Mäder et al., 2021). Currently, the app is able to identify more than 16000 vascular plant species with a focus on the Central European flora. To create an opportunistic plant record, users need to confirm the identification suggested by the classifier. A record consists of the species name, its geolocation, time and date of the observation (Mäder et al., 2021) Since its release in 2018, more than 100 million identification requests have been committed worldwide. In contrast to DWD’s phenology observations, the Flora Incognita records are not following any fixed protocol and are not purposely designed to record phenological data. We refer to this kind of data as “opportunistic”, as they are collected in an unsystematic manner, whenever people are interested to take a picture of a plant to identify it. Due to the AI-based species identification, even botanical laymen without any prior knowledge can identify a plant and provide data. For implementing the same interpolation model for the FI and the DWD data, we used the 2020 and 2021 records of the 17 DWD-based plant species located in Germany, resulting in 491,989 FI records contributing to this study.

### 2.2 Processing Flora Incognita records

#### 2.2.1 Definition of Flora Incognita stations

**Figure 1:**
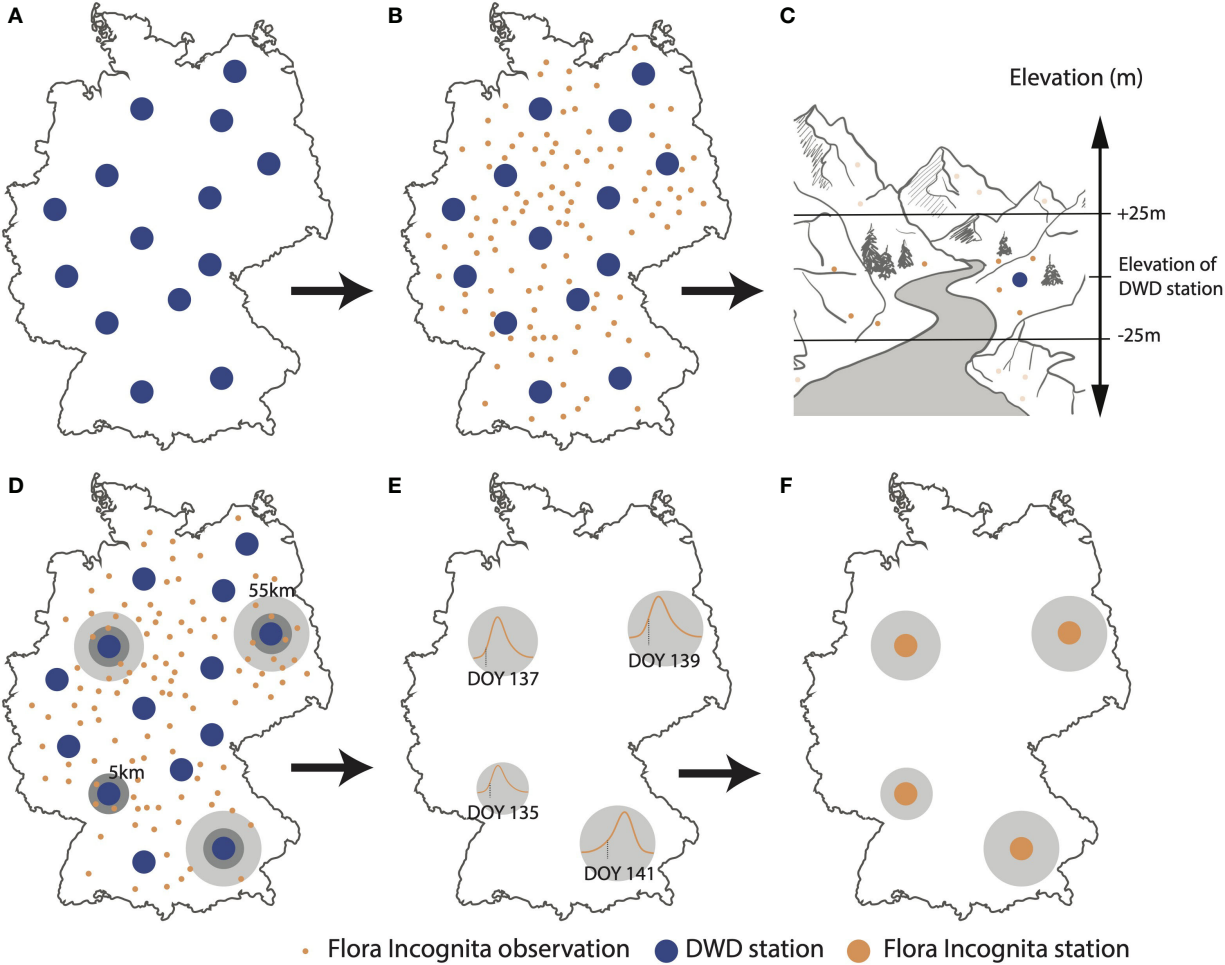
Process diagram, showing how plant observation data are converted into onset of flowering dates and related to DWD (German meteorological service) observation stations for one examplary species. **(A)** Extracting geolocations of DWD stations **(B)** Extracting geolocations of Flora Incognita records for a given year and species. **(C)** Elevation filter: Flora Incognita records in the vicinity of DWD stations are filtered to be in a range of ± 25 m asl. of the elevation of the respective DWD station. **(D)** Radius filter: Flora Incognita observations are filtered based on the distance to the DWD station. Initial radius of 5 km, extendable to max. 55 km until at least 35 observations were present. **(E)** Estimation of the onset of flowering DOY (Day-Of-Year). **(F)** Flora Incognita stations (FI stations) representing onset of flowering DOYs for a subset of the available DWD stations.

In order to use the opportunistic FI observation data for the same phenology interpolation models as the DWD, we defined similar “stations” for the FI data (see **Figure 1**). We selected all FI records of a species within a 5,000m buffer radius and an altitude range of ( ± 25 m asl) around a DWD station, based on the coordinates of all available DWD stations for that species in a given year. This relatively narrow range was chosen due to the significance of altitude as a predictor of phenology and its utilization as an input in the interpolation model. If this number of records reached or exceeded a minimum of 35, the selection process stopped and we used the records within the buffer radius to calculate their day of onset of flowering. If the number of records was lower than 35, the circle’s radius was increased by 1,000 m. This process was iterated until one of the following two conditions was fulfilled: at least 35 records were assigned to that station or its buffer radius exceeded 55,000 m. Records were not necessarily exclusive to only one station. If an observation already contributed to a station while being in the vicinity of another one, it contributed to both stations. If fewer than 35 records were available within a buffer, no station was established at this location.

#### 2.2.2 Calculating the onset of flowering of Flora Incognita observations

In the next step, the number of observations was fitted against their Day-of-Year (DOY) (**Figure 1E**) to illustrate the local distribution of observations throughout the year for each species. By analyzing these observation curves, we determined the onset of flowering. As the functional relationship underlying the onset of flowering is unknown and the curve can have different shapes, we used the R package phenesse (Belitz et al., 2020) to calculate a suitable percentile of the observations curve that represents the onset of flowering for each species. Phenesse uses a parametric bootstrapping approach to calculate phenological metrics for arbitrary percentiles based on the Weibull distribution. As the percentile indicating onset of flowering in a distribution of plant observations from various stages is not obvious, we empirically identified the most appropriate percentile for each species by comparing the median across all gridcells for different percentiles. The percentile closest to the median of the DWD interpolation in 2020 was then chosen as species-specific value and used for both years (**Table 2**). The only exception here was *Galanthus nivalis*, where we could only use the 2021 data as the number of observations in 2020 was too low to define meaningful stations.

**Table 2:** Observed Median (Mdn) and Median absolute deviation (MAD) for the interpolated phenological maps compared across data origin DWD (German meteorological service) and FI (Flora Incognita) in both observed years (2020/2021).

		2020					2021				
		DWD		FI		FI-DWD	DWD		FI		FI-DWD
Species	PCTL	Mdn	MAD	Mdn	MAD	Diff	Mdn	MAD	Mdn	MAD	Diff
*Artemisia vulgaris agg.*	0.55	204	3	205	4	1	208	3	209	4	1
*Fraxinus excelsior*	0.07	–	–	–	–	–	110	5	117	7	7
*Galanthus nivalis*	0.2	–	–	–	–	–	50	4	49	6	1
*Malus sylvestris agg.*	0.2	111	4	113	4	2	126	5	118	6	-8
*Salix caprea*	0.05	67	4	63	7	-4	76	7	62	7	-13
*Sambucus nigra*	0.3	140	6	140	5	0	155	4	153	4	2
*Secale cereale*	0.2	–	–	–	–	–	155	3	157	7	2
*Taraxacum officinale*	0.2	90	6	93	4	3	101	5	102	4	1
*Tilia platyphyllos*	0.35	–	–	–	–	–	173	3	175	7	2
*Tussilago farfara*	0.15	63	4	69	9	6	75	7	68	6	-7

The last column for each year (FI-DWD Diff) shows the differences of the medians between DWD and FI. Onset of flowering in the FI data was estimated as a species-specific percentile (PCTL) of the Weibull distribution.

### 2.3 Spatial interpolation of onset of flowering

Based on the available station data, the DWD employs spatial interpolation techniques to estimate phenology data for each observed stage of various species across Germany. The resulting maps offer a highly resolved spatial representation, providing interpolated Day-Of-Year values for each observed species within 1x1 km grid cells. Our spatial interpolations follow the same procedure as implemented by the DWD, which relies on the longitude, latitude, and elevation of each station location. To achieve this, the administrative area of Germany is divided into overlapping circles with a radius of 1.95°. This division results in a total of 30 circles, with 6 circles in the latitudinal direction and 5 circles in the longitudinal direction. The centers of these circles are also positioned 1.95° apart in both the latitudinal and longitudinal directions.

For each circle we fitted a multiple linear regression based on the onset of flowering dates of the DWD or FI stations:


$$DOY=a_{0}+a_{1}\cdot h+a_{2}\cdot lon+a_{3}\cdot lat$$

where *h* refers to elevation, *lon* refers to longitude and *lat* refers to latitude of the respective station. The corresponding regression coefficients *a*_0_ *,a*_1_ *,a*_2_ and *a*_3_ were attributed to the circle centers and used for inverse distance weighting interpolation for all grid cells, based on all surrounding circles present. The interpolation process is described in more detail by Yuan et al. (2021).

The interpolation was based on the digital terrain model (DGM1000), based on a 1,000 × 1,000m raster file which is publicly available from the Federal Agency for Cartography and Geodesy (https://daten.gdz.bkg.bund.de/produkte/dgm/dgm1000). As almost all DWD stations and most FI stations are located below 1,000m asl we interpolated only grid cells below 1,000m asl, leaving 355,615 grid cells forming the basis for this analysis.

The resulting phenological map was assessed by calculating the root-mean-square errors (RMSE) based on the DWD and FI stations. RMSE is a measure to describe the difference between observed and modelled values and is calculated according to:


$$RMSE=\sqrt{\frac{{\left(y_{obs}-y_{pred}\right)}^{2}}{N_{stn}}}$$

Here, *y_obs_* and *y_pred_* represent observed and modelled values and *N_stn_* is the number of stations. All RMSE values for the DWD-based interpolated maps were lower than 20, so we chose this as a threshold below which we considered interpolations based on FI data as valid in both years. In the following, we refer to values *>* 20 as “high error” and values<=20 as “low error” and only examined the results for the species with an RMSE value below 20. This interpolation routine developed by the DWD depends on a preferably equal distribution of stations across the single circles for which the multiple regressions are carried out. In cases where there are only a limited number of stations within a single circle, the calculated coefficients heavily rely on these few observations. This dependence can lead to abrupt changes in the Day-Of-Year (DOY) values at the boundaries of the circles. This characteristic is an inherent aspect of the interpolation method and can be alleviated by incorporating a larger number of stations. By doing so, the influence of a single station with potentially extreme values on the regression coefficients of a specific circle can be minimized. (see **Figure S3**, *Tussilago farfara* 2020, *Salix caprea* 2020, *Galanthus nivalis* 2021). We still decided to stick to this interpolation method in order to allow full comparability to the interpolation performed by DWD. All analyses, the interpolation and map visualization were performed using the *R* programming language (R Core Team, 2022).

### 2.4 Species characteristics

The 17 selected species are widespread and common in Germany, and it is important to understand why observations of certain species show strong phenological patterns and others don’t: Observations spike when there is a sudden change in attraction to identification app users (i.e. in most cases an attractive and conspicuous flowering phase). As for the targeted phenological observations collected by DWD, the onset of flowering is explicitly recorded for stated plant individuals. In contrast, the probability of a species being identified by app users will depend on certain species characteristics. So we compiled a list of relevant characteristics from the BiolFlor database (Kühn et al., 2004) (**Table 1**): We considered growth form, flowering period, cultivation and conspicuousness as candidates for affecting the probability of observation by identifcation app users. As there is no standard method to evaluate the conspicuousness of a plant we decided to score each species based on our own experience. The basic guideline for this evaluation was whether the species shows an attractive flowering stage that stands out from the non-flowering stage. Three authors (NK, MR and JW) evaluated conspicuousness individually, with disagreements being discussed and collectively resolved afterwards.

## 3 Results

### 3.1 Patterns in the collected plant observations

**Figure 2:**
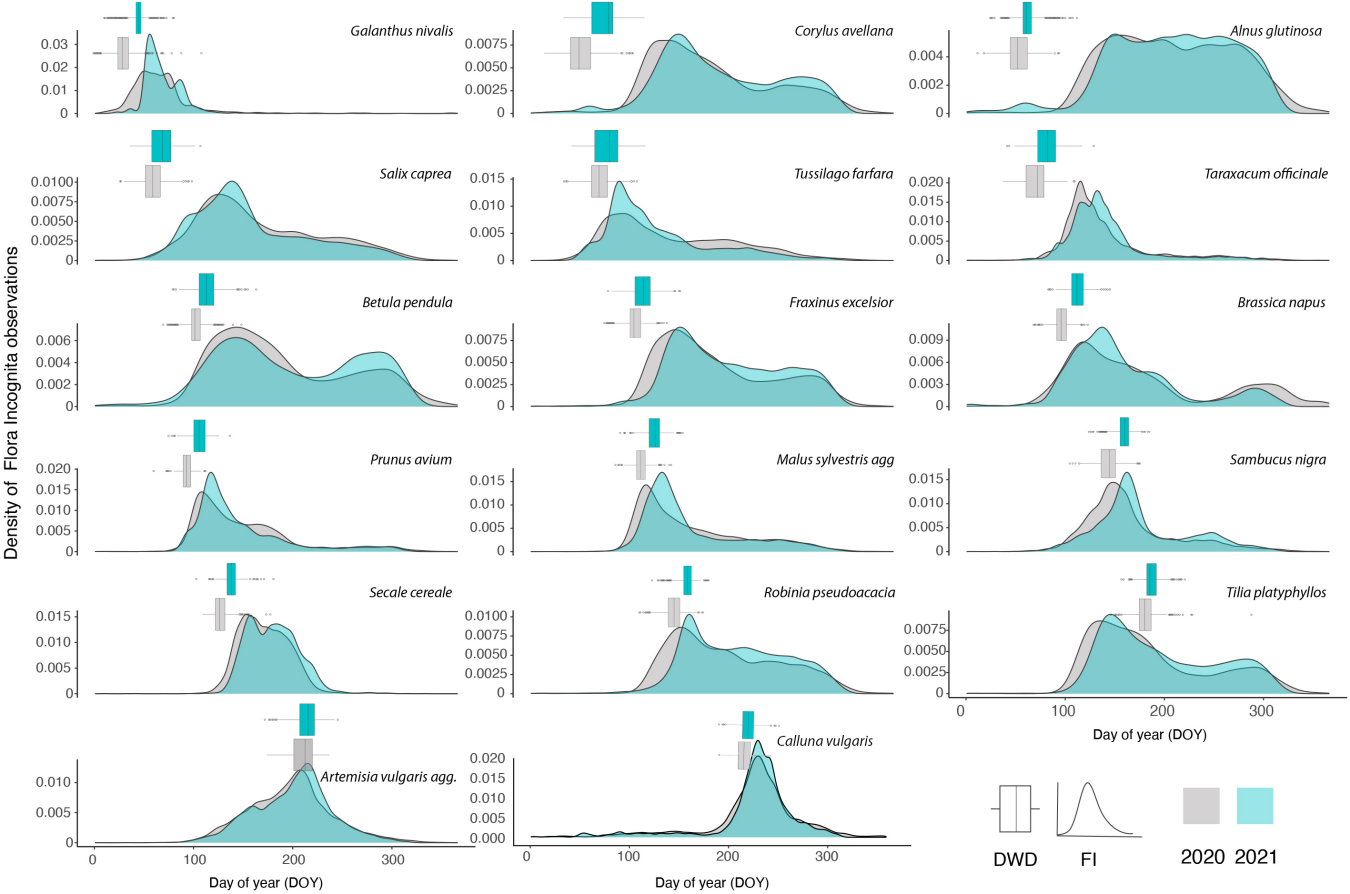
Density of all FI (Flora Incognita) records used in this study collected in 2020 and 2021. The species are ordered along their flowering time. The distribution of DOYs (day of year) for onset of flowering observed across all DWD (German meteorological service) stations are referenced for each species and year in an accompanying boxplot (grey: 2020; blue: 2021). The line in the boxplot represents the median of the distribution.

In the two years, 219,546 (2020) and 207,656 (2021) observations of the 17 species were recorded by Flora Incognita app users (**Table 1**). The number of observations per species ranged between 1,242 (*Galanthus nivalis*; 2020) and 25,878 (*Taraxacum officinale*; 2020), resulting in FI stations ranged between 61 (*Galanthus nivalis*; 2020) and 740 (*Taraxacum officinale*; 2020). The number of DWD stations ranged between 130 (*Prunus avium*; 2021) and 999 (*Galanthus nivalis*; 2021). The majority of FI stations were based on records with a median distance between 25 and 40 km to the center of the station (**Figure S1**). **Figure 2** presents the density of the total Flora Incognita observations per species, and a comparison between both years.

In general, the density of observations follows three different patterns (**Figure 2**). The largest fraction of species shows a single peak during the vegetation season (e.g.,*Taraxacum officinale, Calluna vulgaris, Malus sylvestris*). A number of other species show a more or less pronounced two-peak pattern (e.g., *Betula pendula*, *Brassica napus*). One species (*Alnus glutinosa*) shows a uniform pattern without distinct peaks throughout the growing season. For some species, observation peaks match very closely the onset of flowering observed by DWD (*Prunus avium, Malus sylvestris, Sambucus nigra*). When comparing the same species, the density curves exhibit a high degree of similarity between the two observed years. Conversely, the density curves of different species tend to consistently differ from one another. Examining these density curves reveals that, for most species, observations began earlier in 2020 compared to 2021, aligning with the earlier onset of flowering observed by the DWD stations (**Figure 2**).

### 3.2 Spatial interpolation of onset of flowering

**Figure 3:**
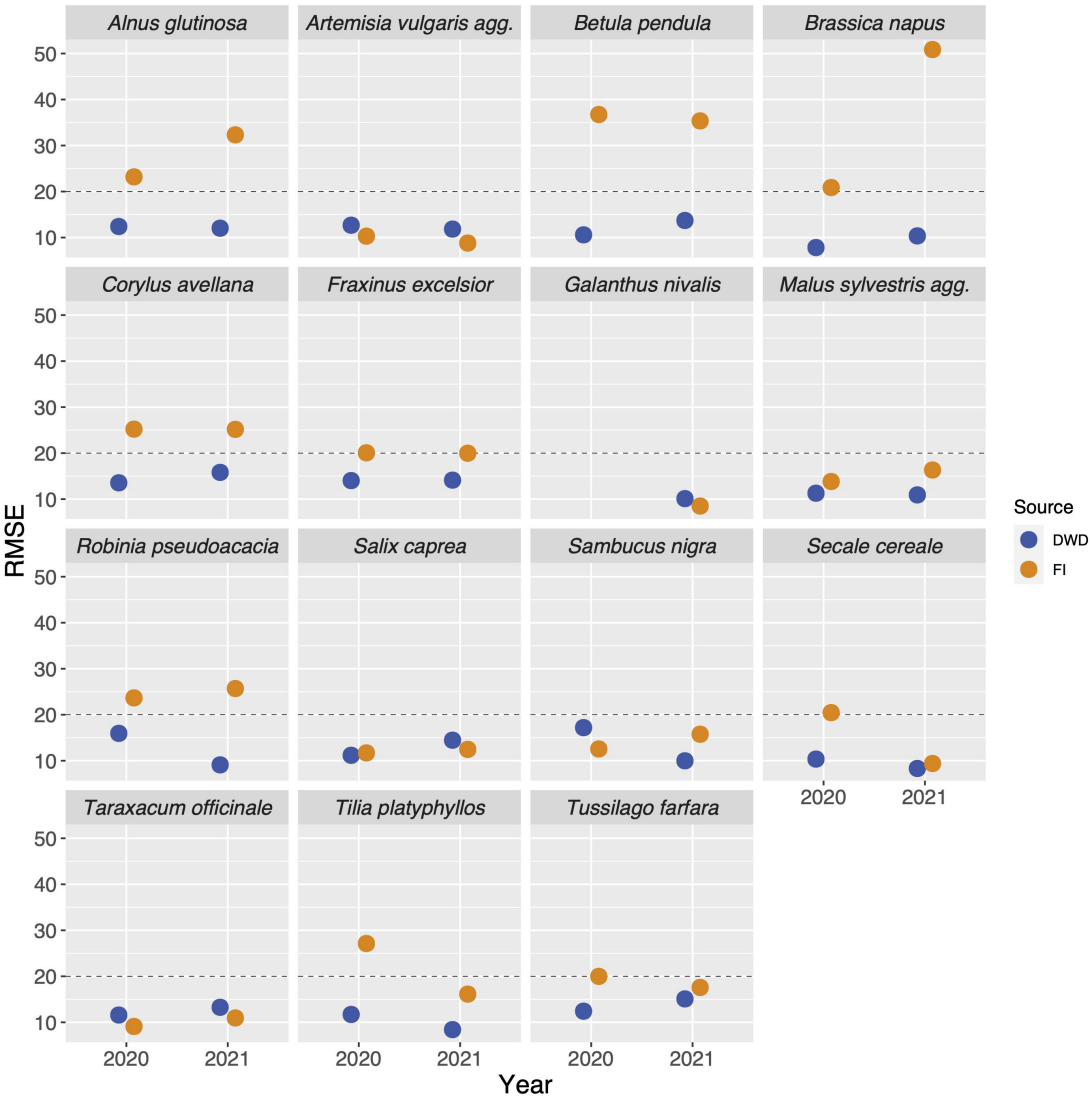
Root-mean-square errors (RMSE) values of the interpolation models compared across data source, year and species. The blue dots represent the RMSE values resulting from the interpolations based on the targeted observations collected by DWD (German meteorological service), the orange dots represent the RMSE values derived from the interpolations based on the opportunistic observations collected by the FI (Flora Incognita) users. We decided to only consider models with RMSE values below 20 (marked with the dashed line) as valid and compared only those to the DWD interpolations (which always yielded values below 20).

**Figure 4:**
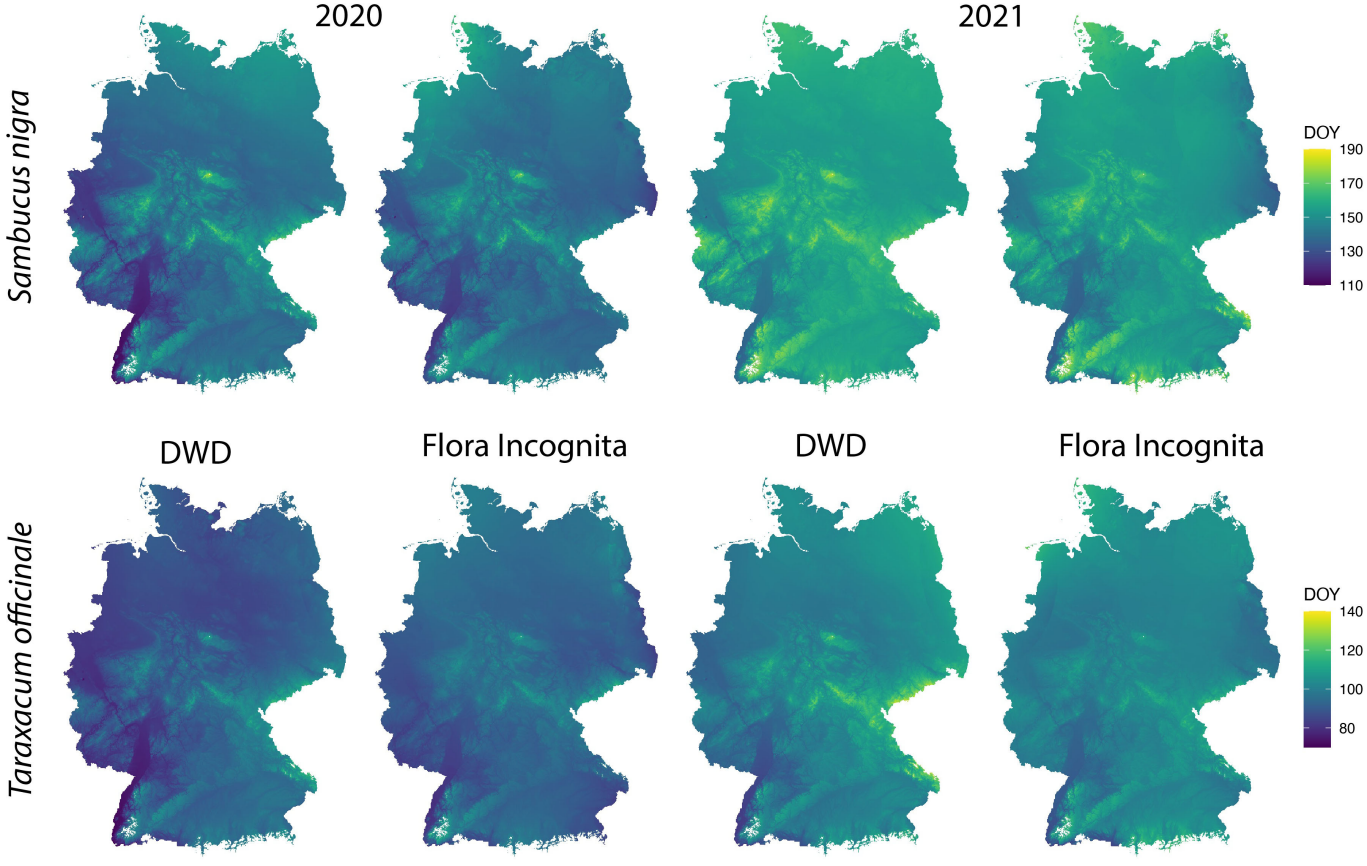
Spatially interpolated maps based on the DWD and FI stations for onset of flowering of *Sambucus nigra* and *Taraxacum officinale* in 2020 and 2021. The colour scale indicates the day of the year of the onset of flowering in each grid cell. The interpolation model is described in more detail in the methods section. Note that the range of colour scales differ between the species.

The percentiles representing onset of flowering as differed between the species and ranged from 0.07 (*Fraxinus excelsior*) to 0.55 (*Artemisia vulgaris*) (**Table 2**) with a majority of values around 0.2. We applied the described interpolation process for the Flora Incognita stations of each species. The calculated RMSE values for 11 out of 17 species were at least in one year below 20 (*Artemisia vulgaris, Galanthus nivalis, Prunus avium, Sambucus nigra, Secale cereale, Malus sylvestris, Tussilago farfara, Salix caprea, Fraxinus excelsior, Tilia platyphyllos* and *Taraxacum officinale*) (**Figure 3**), indicating a comparable quality as the models produced from the DWD stations. In the case of *Artemisia vulgaris, Taraxacum officinale and Galanthus nivalis*, the observed RMSE values for both years are even lower than the RMSE values based on the DWD (see **Figure 3**). However, for some species, the available FI stations were too scarce and/or they were not well distributed across Germany, resulting in some multiple-regression-circles with very few or even without any FI station. To avoid unbalanced predictions, we decided to exclude cases with less than 100 FI stations available (*Calluna vulgaris*, *Prunus avium* and the 2020 observations of *Galanthus nivalis)*. For all remaining species we interpolated maps for different years and for both datasets (DWD + FI), resulting in a total of 32 maps (**Figure S3**). The interpolated maps for *Sambucus nigra* and *Taraxacum officinale* are presented in **Figure 4**. These maps show very similar phenological patterns for the DWD and FI stations within but also between these two years. The onset of flowering of *Sambucus nigra* has occurred 15 days later (median) in 2020 according to the DWD stations and 13 days later according to the Flora Incognita observations **Table 2**. Similarly, the onset of flowering of *Taraxcum officinale* has occurred 11 days later (median) according to DWD and 8 days later according to the Flora Incognita observations. For *Artemisa vulgaris*, both data sources agree that onset of flowering occurred 4 days earlier in 2020. Especially for the two tree species with values from both years, the differences in the medians between both years are much larger *Salix caprea* (DWD: 9 days FI: -1 day), *Malus sylvestris agg.*: (DWD: 15 days FI: 5 days). The differences between the two data sources within the same year are rather low (0-3 days) for *Taraxacum officinale, Sambucus nigra* and *Artemisia vulgaris* but rather large (2-13 days) for *Salix caprea, Tussilago farfara* and *Malus sylvestris* (**Table 2**).

**Figure 5:**
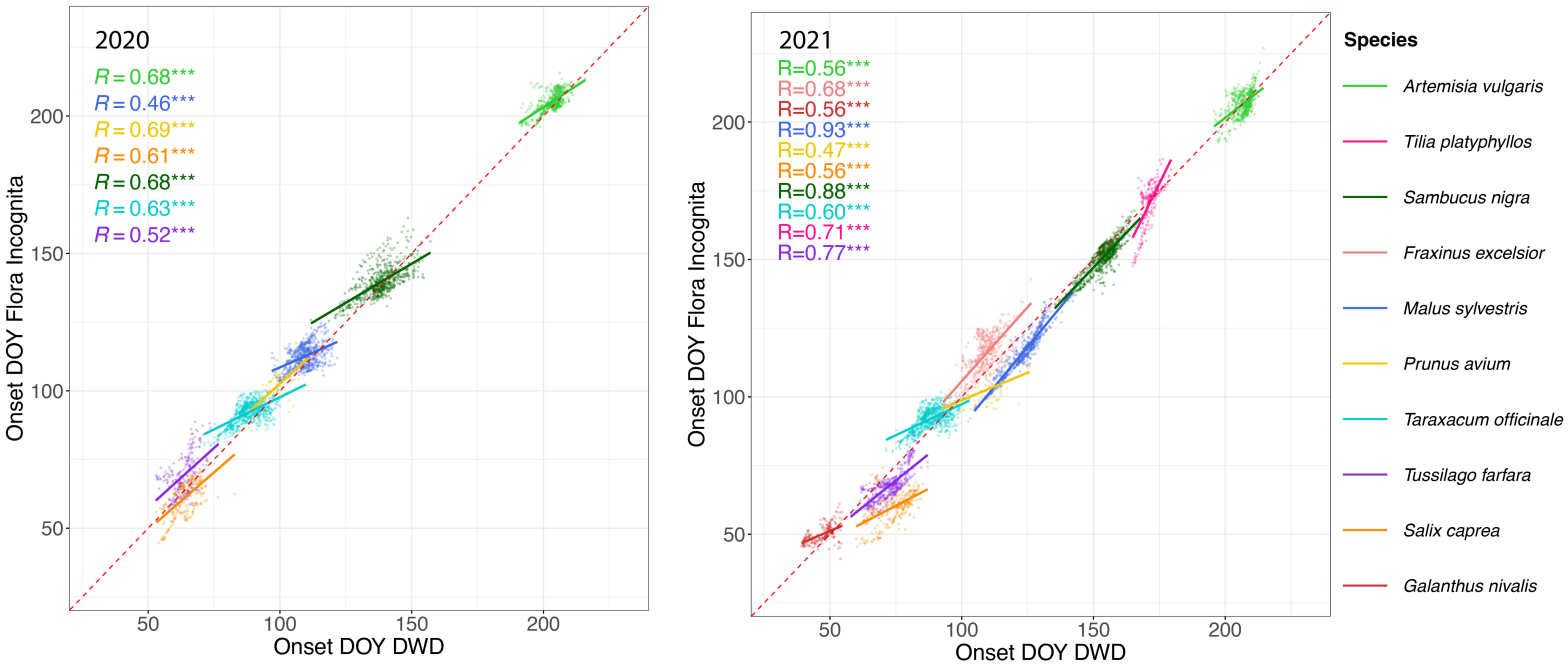
Onset of flowering day of year (DOY) estimated from opportunistic FI (Flora Incognita) records (x-axis) vs. onset of flowering DOY of targeted observations collected by DWD (German meteorological service) (x-axis) in 2020 (left) and 2021 (right). Each dot represents the interpolated DOY for each station with observations from both data sources. Different species are coded by color. Note that the species names in the legend are arranged according to their respective DOY to improve readability The latest flowering species (Artemisia vulgaris) is shown at the top of the legend while the earliest flowering species (Galanthus nivalis) appears at the bottom. The line represents a linear regression conducted separately for each species. The R^2^ values for each linear regression model are shown on the upper left part of each panel. Note that *Galanthus nivalis, Fraxinus excelsior* and *Tilia platyphyllos* are missing in the 2020 panel due to either data scarcity or high RMSE values. *** Indicates p-value ≤ 0.001, suggesting a highly statistically significant correlation.

In general, the estimated values for onset of flowering of the FI stations are highly correlated to onset of flowering observed for the DWD stations (**Figure 5**).

### 3.3 Impact of station count and species traits on interpolation validity

**Figure 6:**
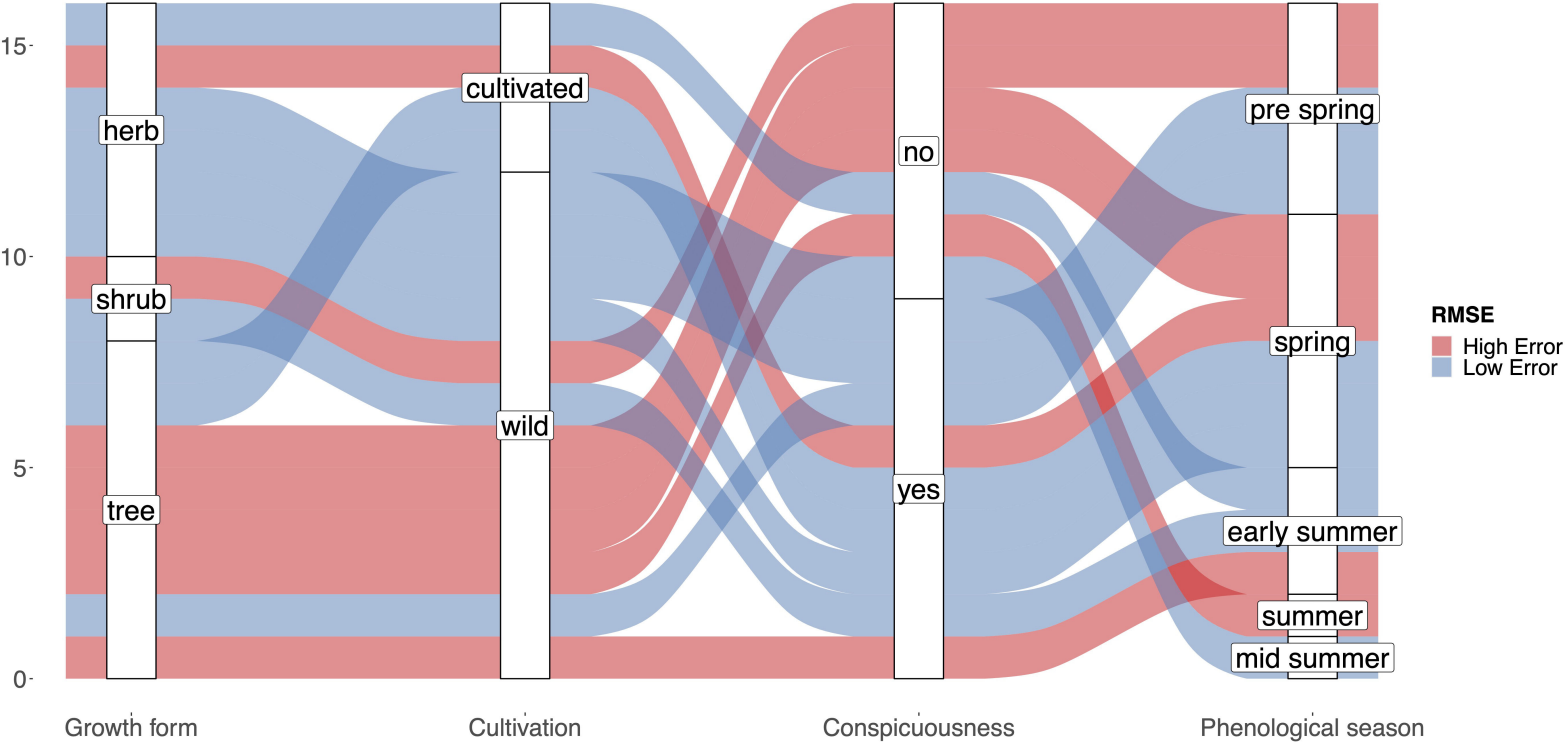
Alluvial plot showing the distribution of plant traits with respect to high/low RMSE values of the resulting interpolation model for each species. The classification of plant traits for each species corresponds to the traits in **Table 1**.

A number of variables might be related to the RMSE of the interpolated model. On the one hand the number of stations may play a role, as fewer stations result in less precise predictions. On the other hand, certain species traits may affect the phenological patterns in opportunistic species observations. The number of available stations was not significantly correlated with the observed RMSE values in any case (DWD (2020): r=0.35; P>0.1; FI (2020): r=0.15; P>0.1, DWD (2021): -0.01; P>0.1, FI (2021): -0.14; P>0.1, see **Figure S2**). Additionally, the collected traits showed rather inconsistent relationships to the quality of the interpolation result (**Figure 6**). Only the growth form “tree” tended to be associated with high RMSE values, as the interpolation of only two out of 7 tree species (Salix caprea and Malus sylvestris) resulted in a valid interpolation. None of the remaining considered traits (cultivation, conspicuousness, phenological season) was associated with low or high RMSE values. Species with valid interpolations are well distributed across the phenological seasons.

## 4 Discussion

In this study, we found that the onset of flowering derived from opportunistic and unsystematic plant observations produce comparable results, at least for certain species, when compared to the data gathered through targeted observations conducted by phenological observers. Especially observations from herbaceous species or shrubs with a conspicious flowering stage *e.g. Sambucus nigra, Taraxacum officinale or Galanthus nivalis* resulted in very similar estimates of onset of flowering as the reference data. However, for certain species, the RMSE values of the calculated estimates of flowering onset were much higher compared to the DWD reference dataset (**Figure 3**) indicating a less precise interpolation. This was especially the case for tree species, where the density curve of the observations already implies that the observations are not linked to the flowering period (**Figure 2**). If onset of flowering is to be estimated from opportunistic plant observations it is necessary to identify the suitable percentile in the observation curve that represents the flowering stage. In the current analysis we deduced the most suitable percentile per species based on a comparison to the median of the actual DWD estimates in 2020 if present. We always used the same percentile for the 2021 estimates and found very similar results for the 2021 interpolations. Here we assume a consistent, species-specific pattern of observations throughout the year which is mainly affected by phenological differences between the years (**Figure 2**). Once longer time series of opportunistic observation data have been accumulated it would be possible to better validate these species-specific values. An alternative approach would be to use the same percentile for all species (e.g. 50th percentile i.e. median observation date). The resulting estimate would be equally well suited to represent a robust means to enable inter-species phenological comparisons. However, it cannot be directly linked to the onset of the flowering. Based on the relative timing of flowering compared to the overall observation curve, we found different percentiles useful for different species. For example the percentile for *Salix caprea* or *Fraxinus excelsior* are lower than 0.1 These species are an example of trees flowering prior to leaf-out very early in the year at the edge of their overall observation curves. For most of the herb or shrub species, a percentile of 0.2 represented the onset of flowering. *Artemisa vulgaris* is an exception here. This species is a herbaceous, perennial plant that is often found growing on roadsides, field margins and uncultivated areas in urban context, where it starts growing in early spring and reaches heights of up to 2m. Therefore, *Artemisia vulgaris* may attract peoples’ interest already through its dominant appearance which is only slightly changed with the onset of flowering of the inconspicuous, wind-pollinated flower heads. Yet, we explicitly decided to choose the same set of species that is observed by the German Meteorological Service to allow a direct comparison of the interpolated products based on the different data sources.

In general, the interpolated maps based on the opportunistic plant records capture the differences in onset of flowering between years in many cases very similar to the targeted observations (e.g., *Taraxacum officinale, Sambucus nigra, Artemisia vulgaris*). However, the median estimate for *Tussilago farfara* was 6 days earlier in 2020 but 7 days later in 2021 when compared to the reference observations form DWD. The overall observation density distribution curve for *Tussilago farfara* (**Figure 2**) shows a different pattern for 2020 and 2021. The peak during flowering time around DOY 100 is much less pronounced compared to 2021 while the relative fraction of observations of the later stages is higher in 2020. Additionally, the number of observations in 2020 is much lower (7,945) compared to the number of observations in 2021 (13,391), resulting in only half the number of FI Stations in 2020 (**Table 1**). The most likely explanation for these different patterns is the first Covid lockdown in Germany lasting from DOY 82 to 125. This may have resulted in a reduced number of observations during peak flowering time of *Tussilago farfara* thereby increasing the relative fraction of observations collected at the later stages when the lockdown was lifted. The resulting different shapes of the observation curves will affect the estimates of the 0.15 percentiles of the curves and lead to the observed discrepancies. This implies a certain degree of caution, as overall societal events suddenly affecting the behavior of people may influence the estimates of phenology. The focus on trees and cultivated plants is useful in national monitoring programs, as it allows to observe an individual tree over a longer time period, longer than a single year. But tree species often do not exhibit the key traits that makes them suitable for opportunistic phenology monitoring (**Figure 6**). The observed number of species in this study is not sufficient to identify the most suitable set of traits that characterize plants as susceptible for phenology monitoring via app observations. However, Our results showed that suitable species are typically abundant, herbaceous and have only a single, temporarily limited and prominent flowering stage (**Figure 6**). This is in line with observations by Puchałka et al. (2022). These authors found that unstructured citizen science data can be a reliable source allowing to develop a model of plant phenology for *Anemone nemorosa*. The authors state that they could conduct their approach so successfully because there were many observations available for *Anemone nemorosa*, for it is a common spring flower that people often search for and document using citizen science apps. Opportunistic, crowdsourced plant images have also been successfully used to detect unusual flowering events in *Yucca* (Barve et al., 2020) and to identify drivers of flowering duration (Li et al., 2021). Based on our results we suggest that unsystematic, opportunistic plant observation can be used as input for the interpolation models in a similar way as systematic phenological observations. The onset of flowering estimates based on such opportunistic plant observations could be easily integrated in annual phenology monitoring schemes and complement the systematic observations, thereby filling the data gaps created by the decline in phenology observers. However, unlike systematic observations, they cannot be directly used as data points, because the phenological stage of a single observation is unknown. Instead, opportunistic data need to be prepared in a way that allows comparison. Our example uses the estimation of the onset of flowering for a larger sample of observations within a limited distance to the location and a suitable elevation range. We used a minimum of 35 observations per location to estimate onset of flowering which represents a compromise between accuracy and coverage. Still this resulted in insufficient coverage of some regions for some species with few occurrences (e.g. *Brassica napus, Calluna vulgaris*) and ultimately to their exclusion due to data scarcity. The approach used here was tailored towards the direct comparison against systematically collected reference data. For other purposes, it is also possible to use a grid cell raster and estimate onset of flowering for each gridcell separately.

### 4.1 Caveats

Both datasets are influenced by inherent biases that might affect the resulting interpolations. The number of FI observations strongly correlates to the populated area in a specific region (Mahecha et al., 2021). Consequently, more observations are collected in densely populated urban areas, which also tend to be connected with higher air temperatures (Wohlfahrt et al., 2019; Li et al., 2021) and in consequence may affect the flowering phenology of the observed plants. However, it is likely that, although to a lesser degree, the location of the DWD stations are prone to the same bias. In general, both datasets had relatively scarce covering of areas at higher elevations (Yuan et al., 2021), which will lead to more considerable uncertainties in the interpolation at higher elevations. Since we created FI stations based on the locations of existing DWD stations, we avoided some of these biases and related problems and made both datasets as comparable as possible. The number of observations was not directly linked to the RMSE of the resulting interpolation in FI data (see **Figure S3**), a result that was also observed for the DWD data by Yuan et al. (2021).

### 4.2 Perspectives

Opportunistic plant observations represent a massively increasing and powerful source of data (Moles and Xirocostas, 2022). Despite the biases and challenges discussed above, such data provide a unique chance to complement targeted observations collected by trained observers from phenological monitoring networks and also make a crucial long-term contribution in measuring phenological shifts and investigating their impact on the structure and functioning of ecosystems (Iwanycki Ahlstrand et al., 2022). However, our approach does not work well for all species to the same extent.

Species that are suitable for phenological monitoring based on opportunistic observations often exhibit different traits than species that are monitored by phenological observers of the DWD so far. While e.g. trees are an important cornerstone in the phenological monitoring program of the DWD, most trees have proven rather unsuitable in the current study (**Figure 5**). As phenological observers can repeatedly visit the same tree and observe buds and flowers to determine the onset of leaf folding or flowering, specific phenological stages of widespread trees in Germany often do not attract the eye and interest of identification app users. Therefore, although high numbers of observations are present for many tree species (**Table 1**), the observation dates are often not clearly linked to a specific phenological stage and are rather constant during the vegetation season. An example would be *Alnus glutinosa*, which shows multi-peak patterns or follows a pattern with less pronounced peaks stretching over longer time periods (**Figure 2**). However, it is noteworthy that the shape of the observation density curves are on the one hand often very different between species, but on the other hand remarkably similar across the two observed years for the same species (**Figure 2**). This suggests a consistent, species-specific, sometimes phenology-related observation pattern that can be utilized using new approaches. Here we chose to estimate the onset of flowering based on the species observations in order to directly compare it with longer time series such as the DWD data. However, the mean observation date usually represents a more robust estimate for opportunistic plant observation data (de Keyzer et al., 2017; Iwanycki Ahlstrand et al., 2022). Therefore, comparing time series only covering opportunistic plant observations, using the peak of observation could potentially yield more robust results. But comparing methods for the robustness of the flowering date estimates was not the focus of this study.

One logical next step would be to additionally analyze the images taken by the users during the identification process, to recognize different phenological stages directly on the image. However, as shown in earlier attempts in previous studies with citizen science data, this involves time-consuming, manual annotation, e.g., whether a flower is present or not (Barve et al., 2020).

A first published study shows that deep learning algorithms can be used to successfully extract phenological information from citizen science images (Reeb et al., 2022). They used a convolutional neuronal network to classify *Alliaria petiolata* images into different phenological stages. In the first step for a two-stage phenology (flowering and non-flowering), they reached 95.9% accuracy while in the second step with four-stage phenology (vegetative, budding, flowering, and fruiting), they reached 86.4%. Another study conducted image-based monitoring of field plots using near-surface time-lapse cameras, subsequent automated detection and counting of flowers in the images by using a convolutional neural network. This study also shows that automatic identification of phenological stages of two Arctic species (*Dryas octopetala* and *Dryas integrifolia*) is possible. In the long term, applying these techniques to opportunistically collected images might contribute to fine-scale phenological monitoring. Moreover, phenophases such as fruitification, leaf onset, and senescence can also be detected and analyzed, which allows analyses of multiple phenological phases at the same time and on smaller scales. Last but not least, phenological phases, inferred from crowd-sourced data could help to validate plant phenology from remote sensing and will be used to parametrize phenological models (Puchałka et al., 2022). It would be an important task for the future to identify plant species that are suitable for phenological monitoring, especially since the number of crowd-sourced and opportunistic data will continue to increase in the future. As many citizen-science-based phenological monitoring networks such as DWD suffer from decreasing numbers of participants (Yuan et al., 2021), followed by reduced coverage of the phenological observations, utilizing crowd-sourced plant observation data of suitable species could help to compensate the loss of phenological observers.

## 5 Conclusions

Extracting phenological patterns from opportunistic species observations may compensate for the ongoing loss of observers in phenological citizen science networks. We argue that opportunistic plant observations could easily be integrated into existing phenology monitoring efforts, extending the set of monitored species to those that are frequently observed by identification app users, therewith providing more fine-grained phenological data. By harnessing unstructured and opportunistic data in this manner, we can make a valuable contribution to quantifying phenological shifts associated with ongoing climatic changes.

## Data availability statement

The raw data supporting the conclusions of this article will be made available by the authors, without undue reservation.

## Author contributions

MR and JW conceived the idea; MR and NK designed methodology; JW, CR, and PM gave comments to the methodology; TM programming the function to select the FI -stations; NK and MR analyzed the data; NK, MR, and JW visualized the data; MR and NK led the writing of the manuscript, JW participated throughout the whole writing process; PM, CW, DB, MR, and JW developed the Flora Incognita app, provided and preprocessed the data; JW and PM were responsible for funding acquisition. All authors contributed to the article and approved the submitted version.

## Supplementary material

Supplementary Figure 1 Distribution of the buffer radii of FI stations for observed species in 2020 and 2021.

Supplementary Figure 2 RMSE in relation to the number of observation stations for DWD (blue) and Flora incognita observations (orange). DWD (2020): r=0.35; P*>*0.1; FI (2020): r=0.15; P*>*0.1, DWD (2021): -0.01; P*>*0.1, FI (2021): -0.14; P*>*0.1.

Supplementary Figure 3 Spatially interpolated maps for eight species based on the DWD and FI stations for onset of flowering in year 2020 and 2021.

## References

[B1] Badeck F. Bondeau A. Bottcher K. Doktor D. Lucht W. Schaber J.. (2004). Responses of spring phenology to climate change. New Phytol. 162, 295–309. doi: 10.1111/j.1469-8137.2004.01059.x

[B2] Barve V. V. Brenskelle L. Li D. Stucky B. J. Barve N. V. Hantak M. M.. (2020). Methods for broad-scale plant phenology assessments using citizen scientists photographs. Appl. Plant Sci. 8, e11315. doi: 10.1002/aps3.11315 31993257 PMC6976896

[B3] Beaubien E. G. Hamann A. (2011). Plant phenology networks of citizen scientists: recommendations from two decades of experience in Canada. Int. J. Biometeorology 55, 833–841. doi: 10.1007/s00484-011-0457-y 21688202

[B4] Belitz M. W. Larsen E. A. Ries L. Guralnick R. P. (2020). The accuracy of phenology estimators for use with sparsely sampled presence-only observations. Methods Ecol. Evol. 11, 1273–1285. doi: 10.1111/2041-210X.13448

[B5] Berra E. F. Gaulton R. (2021). Remote sensing of temperate and boreal forest phenology: A review of progress, challenges and opportunities in the intercomparison of *in-situ* and satellite phenological metrics. For. Ecol. Manage. 480, 118663. doi: 10.1016/j.foreco.2020.118663

[B6] Bonnet P. Joly A. Faton J. Brown S. Kimiti D. Deneu B.. (2020). How citizen scientists contribute to monitor protected areas thanks to automatic plant identification tools. Ecol. Solutions Evidence 1, 1–18. doi: 10.1002/2688-8319.12023

[B7] Brown C. J. O’Connor M. I. Poloczanska E. S. Schoeman D. S. Buckley L. B. Burrows M. T.. (2016). Ecological and methodological drivers of species’ distribution and phenology responses to climate change. Global Change Biol. 22, 1548–1560. doi: 10.1111/gcb.13184 26661135

[B8] Bucher S. F. König P. Menzel A. Migliavacca M. Ewald J. Römermann C. (2018). Traits and climate are associated with first flowering day in herbaceous species along elevational gradients. Ecol. Evol. 8, 1147–1158. doi: 10.1002/ece3.3720 29375786 PMC5773311

[B9] Cleland E. Chuine I. Menzel A. Mooney H. Schwartz M. (2007). Shifting plant phenology in response to global change. Trends Ecol. Evol. 22, 357–365. doi: 10.1016/j.tree.2007.04.003 17478009

[B34] Core Team R. (2022). R: A language and environment for statistical computing (Vienna, Austria: R Foundation for Statistical Computing).

[B10] de Keyzer C. W. Rafferty N. E. Inouye D. W. Thomson J. D. (2017). Confounding effects of spatial variation on shifts in phenology. Global Change Biol. 23, 1783–1791. doi: 10.1111/gcb.13472 27550575

[B11] Denny E. G. Gerst K. L. Miller-Rushing A. J. Tierney G. L. Crimmins T. M. Enquist C. A. F.. (2014). Standardized phenology monitoring methods to track plant and animal activity for science and resource management applications. Int. J. Biometeorology 58, 591–601. doi: 10.1007/s00484-014-0789-5 PMC4023011 24458770

[B13] Goodfellow I. Bengio Y. Courville A. (2016). Deep learning (Cambridge, MA, United States: MIT Press).

[B14] Inouye D. W. (2022). Climate change and phenology. WIREs Climate Change 13, e764. doi: 10.1002/wcc.764

[B15] Iwanycki Ahlstrand N. Primack R. B. Tøttrup A. P. (2022). A comparison of herbarium and citizen science phenology datasets for detecting response of flowering time to climate change in Denmark. Int. J. Biometeorology 66, 849–862. doi: 10.1007/s00484-022-02238-w PMC9042978 35235036

[B16] Jones H. G. (2020). What plant is that? tests of automated image recognition apps for plant identification on plants from the british flora. AoB Plants 12, plaa052. doi: 10.1093/aobpla/plaa052 33173573 PMC7640754

[B17] Kaspar F. Zimmermann K. Polte-Rudolf C. (2014). An overview of the phenological observation network and the phenological database of Germany’s national meteorological service (deutscherwetterdienst). Adv. Sci. Res. 11, 93–99. doi: 10.5194/asr-11-93-2014

[B18] Katal N. Rzanny M. Mäder P. Wäldchen J. (2022). Deep learning in plant phenological research: A systematic literature review. Front. Plant Sci. 13. doi: 10.3389/fpls.2022.805738 PMC8969581 35371160

[B19] Koch E. Bruns E. Chmielewski F.-M. Defila C. Lipa W. Menzel A. (2007). Guidelines for plant phenological observations. World Climate Data Monit. Programme.

[B20] Konig P. Tautenhahn S. Cornelissen J. H. C. Kattge J. Bonisch G. Römermann C. (2018). Advances in flowering phenology across the northern hemisphere are explained by functional traits. Global Ecol. Biogeography 27, 310–321. doi: 10.1111/geb.12696

[B21] Kühn I. Durka W. Stefan K. (2004). Biolflor — a new plant-trait database as a tool for plant invasion ecology. Diversity Distributions 10, 363–365. doi: 10.1111/j.1366-9516.2004.00106.x

[B22] Li D. Barve N. Brenskelle L. Earl K. Barve V. Belitz M. W.. (2021). Climate, urbanization, and species traits interactively drive flowering duration. Global Change Biol. 27, 892–903. doi: 10.1111/gcb.15461 33249694

[B23] Mäder P. Boho D. Rzanny M. Seeland M. Wittich H. C. Deggelmann A.. (2021). The flora incognita app – interactive plant species identification. Methods Ecol. Evol. 12, 1335–1342. doi: 10.1111/2041-210X.13611

[B24] Mahecha M. D. Rzanny M. Kraemer G. Mäder P. Seeland M. Wäldchen J. (2021). Crowdsourced plant occurrence data provide a reliable description of macroecological gradients. Ecography 44, 1131–1142. doi: 10.1111/ecog.05492

[B25] Moles A. T. Xirocostas Z. A. (2022). Statistical power from the people. Nat. Ecol. Evol 6(12), 1802–1803. doi: 10.1038/s41559-022-01902-z 36266457

[B26] Montgomery R. A. Rice K. E. Stefanski A. Rich R. L. Reich P. B. (2020). Phenological responses of temperate and boreal trees to warming depend on ambient spring temperatures, leaf habit, and geographic range. Proc. Natl. Acad. Sci. 117, 10397–10405. doi: 10.1073/pnas.1917508117 32341148 PMC7229751

[B27] Morisette J. T. Richardson A. D. Knapp A. K. Fisher J. I. Graham E. A. Abatzoglou J.. (2009). Tracking the rhythm of the seasons in the face of global change: phenological research in the 21st century. Front. Ecol. Environ. 7, 253–260. doi: 10.1890/070217

[B28] Müller-Westermeier G. (1995). Numerisches Verfahren zur Erstellung klimatologischer Karten. Reports of the Deutscher Wetterdienst 193 Deutscher Wetterdienst, Offenbach.

[B30] Nordt B. Hensen I. Bucher S. F. Freiberg M. Primack R. B. Stevens A.-D.. (2021). The phenobs initiative: A standardised protocol for monitoring phenological responses to climate change using herbaceous plant species in botanical gardens. Funct. Ecol. 35, 821–834. doi: 10.1111/1365-2435.13747

[B500] National Phenology Network. (2022). National phenology network (usa-npn). Available at: https://www.usanpn.org (Accessed 2022-11-16).

[B31] Pärtel J. Pärtel M. Wäldchen J. (2021). Plant image identification application demonstrates high accuracy in northern europe. AoB Plants 13, plab050. doi: 10.1093/aobpla/plab050 34457230 PMC8387968

[B32] Piao S. Liu Q. Chen A. Janssens I. A. Fu Y. Dai J.. (2019). Plant phenology and global climate change: Current progresses and challenges. Global Change Biol. 25, 1922–1940. doi: 10.1111/gcb.14619 30884039

[B33] Puchałka R. Klisz M. Koniakin S. Czortek P. Dylewski Łukasz Paz-Dyderska S.. (2022). Citizen science helps predictions of climate change impact on flowering phenology: A study on anemone nemorosa. Agric. For. Meteorology 325, 109133. doi: 10.1016/j.agrformet

[B35] Reeb R. A. Aziz N. Lapp S. M. Kitzes J. Heberling J. M. Kuebbing S. E. (2022). Using convolutional neural networks to efficiently extract immense phenological data from community science images. Front. Plant Sci. 3148. doi: 10.3389/fpls.2021.787407 PMC8801702 35111176

[B36] Renner S. S. Chmielewski F.-M. (2022). The international phenological garden networto 2021): its 131 gardens, cloned study species, data archiving, and future. Int. J. Biometeorology 66, 35–43. doi: 10.1007/s00484-021-02185-y PMC8727390 34491440

[B37] Richardson A. D. (2019). Tracking seasonal rhythms of plants in diverse ecosystems with digital camera imagery. New Phytol. 222, 1742–1750. doi: 10.1111/nph.15591 30415486

[B38] Richardson A. D. Keenan T. F. Migliavacca M. Ryu Y. Sonnentag O. Toomey M. (2013). Climate change, phenology, and phenological control of vegetation feedbacks to the climate system. Agric. For. Meteorology 169, 156–173. doi: 10.1016/j.agrformet.2012.09.012

[B39] Taylor S. D. Meiners J. M. Riemer K. Orr M. C. White E. P. (2019). Comparison of largescale citizen science data and long-term study data for phenology modeling. Ecology 100, e02568. doi: 10.1002/ecy.2568 30499218 PMC7378950

[B40] van Vliet A. J. H. de Groot R. S. Bellens Y. Braun P. Bruegger R. Bruns E.. (2003). The european phenology network. Int. J. Biometeorology 47, 202–212. doi: 10.1007/s00484-003-0174-2 12734744

[B41] Villon S. Mouillot D. Chaumont M. Subsol G. Claverie T. Villeger,´ S. (2020). A new method to control error rates in automated species identification with deep learning algorithms. Sci. Rep. 10, 1–13. doi: 10.1038/s41598-020-67573-7 32620873 PMC7334229

[B44] Wäldchen J. Mäder P. (2018). Machine learning for image based species identification. Methods Ecol. Evol. 9, 2216–2225. doi: 10.1111/2041-210X.13075

[B42] White M. A. de BEURS K. M. Didan K. Inouye D. W. Richardson A. D. Jensen O. P.. (2009). Intercomparison, interpretation, and assessment of spring phenology in North America estimated from remote sensing for 1982-2006. Global Change Biol. 15, 2335–2359. doi: 10.1111/j.1365-2486.2009.01910.x

[B43] Wohlfahrt G. Tomelleri E. Hammerle A. (2019). The urban imprint on plant phenology. Nat. Ecol. Evol. 3, 1668–1674. doi: 10.1038/s41559-019-1017-9 31712692 PMC6882677

[B45] Yuan Y. Harer S. Ottenheym T. Misra G. Lüpke A. Estrella N.. (2021). Maps, trends, and temperature sensitivities–phenological information from and for decreasing numbers of volunteer observers. Int. J. Biometeorology 65, 1377–1390. doi: 10.1007/s00484-021-02110-3 PMC8346396 33694098

[B46] Zimmermann K. Polte-Rudolf C. (2013). Prüfung und Korrektur phänologischer Daten. Phanologie- J. 41.

