# Supplementary figures and images for: Bridging the gap: how to adopt opportunistic plant observations for phenology monitoring
Source: Front Plant Sci. 2023 Oct 4;14:1150956. doi: 10.3389/fpls.2023.1150956 (PMC10582721; doi:10.3389/fpls.2023.1150956)

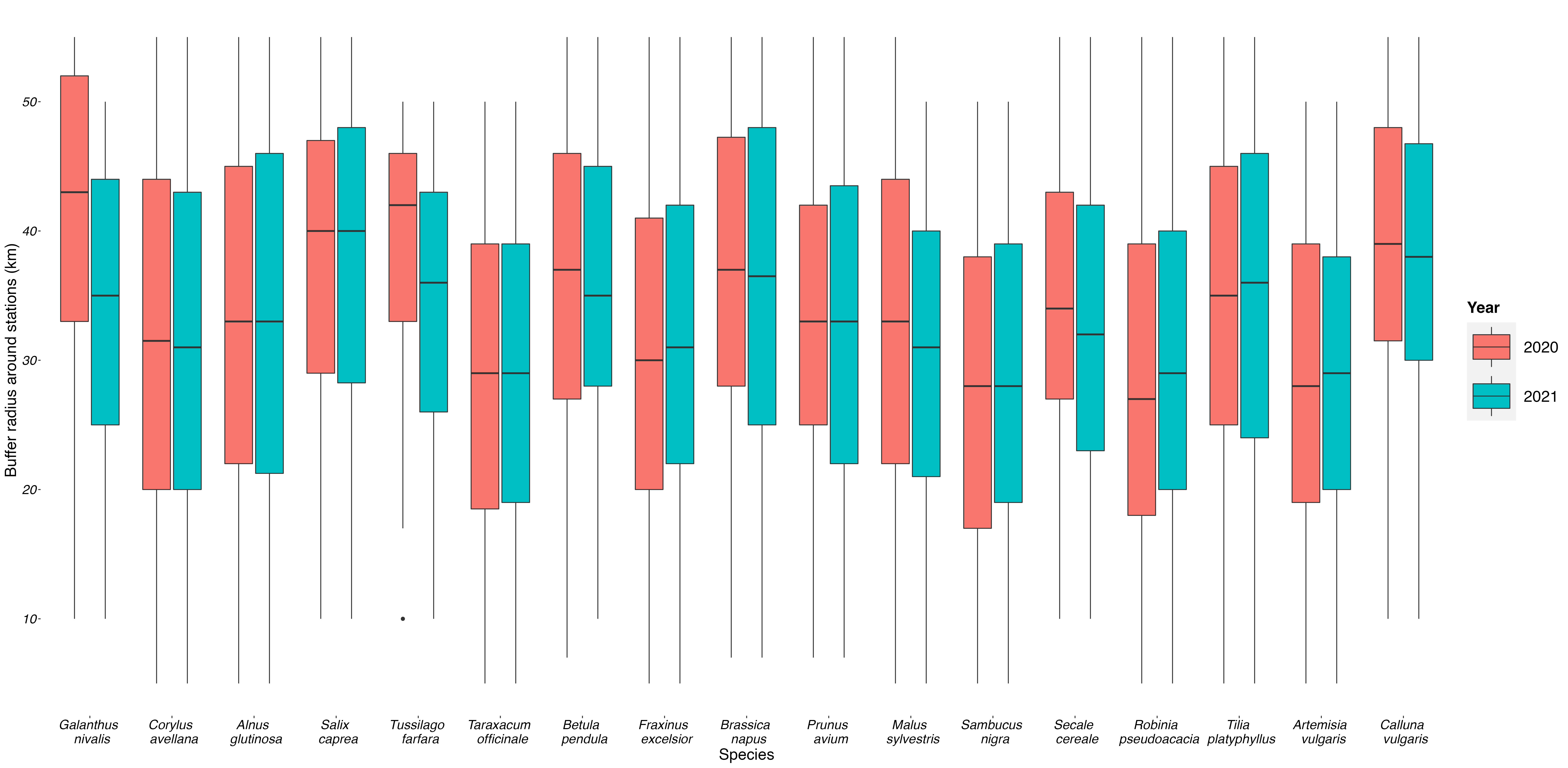

Supplement: Supplementary Figure 1 — Distribution of the buffer radii of FI stations for observed species in 2020 and 2021. [file Image_1.jpeg]

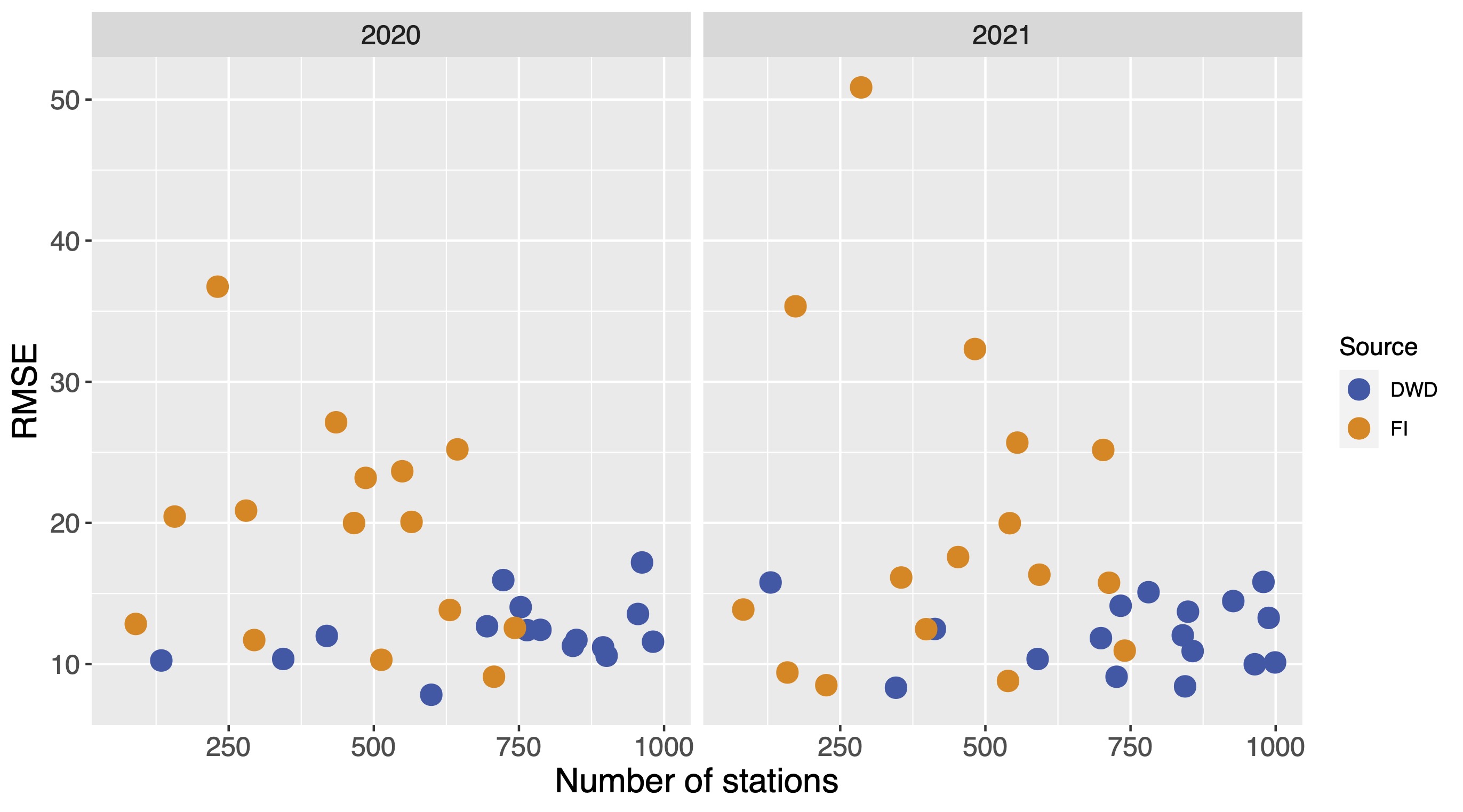

Supplement: Supplementary Figure 2 — RMSE in relation to the number of observation stations for DWD (blue) and Flora incognita observations (orange). DWD (2020): r=0.35; P>0.1; FI (2020): r=0.15; P>0.1, DWD (2021): -0.01; P>0.1, FI (2021): -0.14; P>0.1. [file Image_2.jpeg]

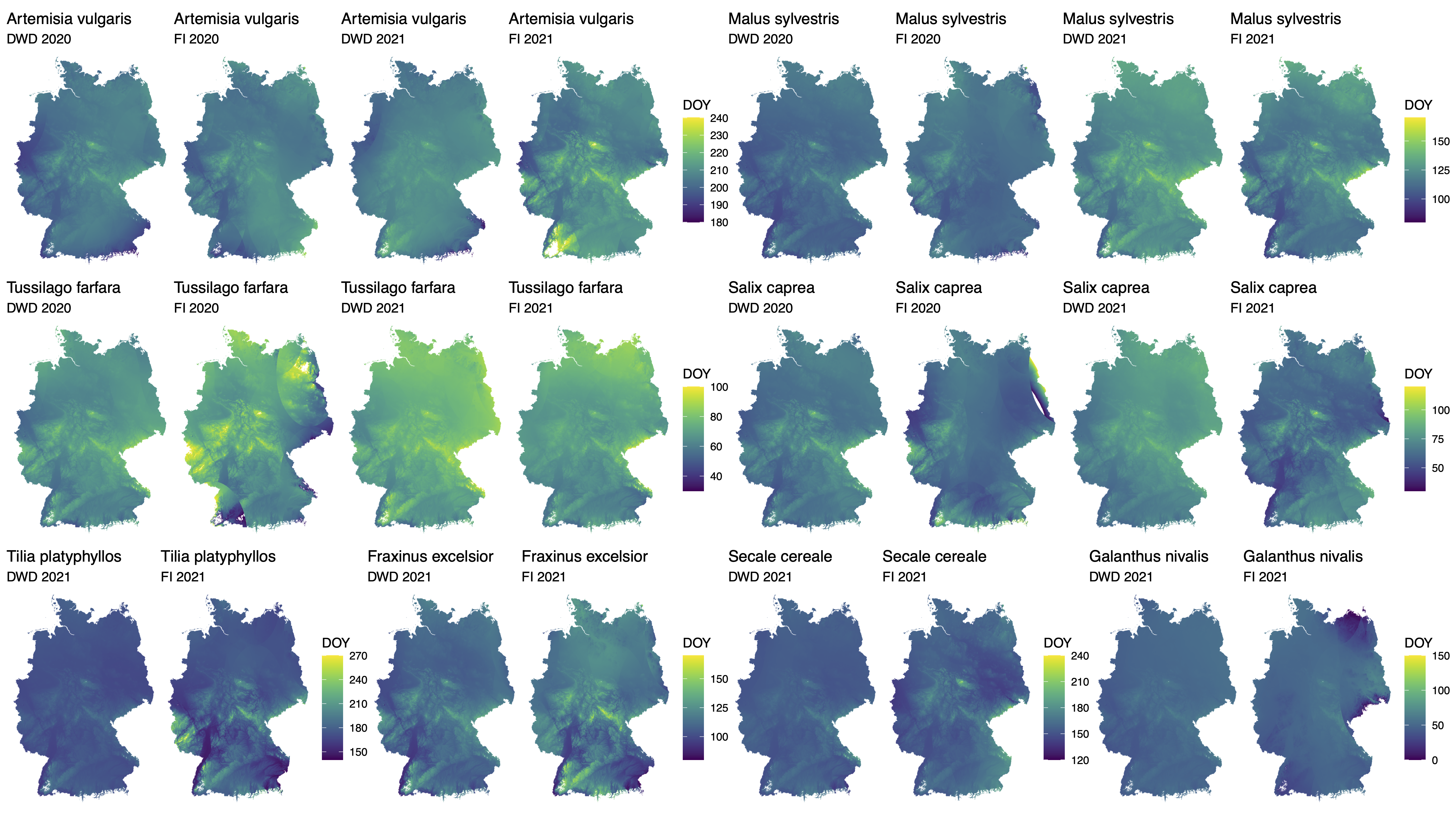

Supplement: Supplementary Figure 3 — Spatially interpolated maps for eight species based on the DWD and FI stations for onset of flowering in year 2020 and 2021. [file Image_3.jpeg]
